# Nomogram predicting leukopenia in osteosarcoma after high-dose methotrexate chemotherapy

**DOI:** 10.18632/aging.203978

**Published:** 2022-05-31

**Authors:** Haixiao Wu, Guijun Xu, Zhijun Li, Yao Xu, Yile Lin, Vladimir P. Chekhonin, Karl Peltzer, Jun Wang, Shu Li, Huiyang Li, Jin Zhang, Yuan Xue, Wenjuan Ma, Xin Wang, Chao Zhang

**Affiliations:** 1 Tianjin Medical University Cancer Institute and Hospital, National Clinical Research Center for Cancer, Key Laboratory of Cancer Prevention and Therapy, Tianjin’s Clinical Research Center for Cancer, Tianjin, China; 2 The Sino-Russian Joint Research Center for Bone Metastasis in Malignant Tumor, Tianjin, China; 3 Department of Orthopedics, Tianjin Hospital, Tianjin University, Tianjin, China; 4 Department of Health Management Center (Epidemiology and Biostatistics), First Affiliated Hospital, Army Medical University, Chongqing, China; 5 Department of Orthopaedic Surgery, Tianjin Medical University General Hospital, Tianjin, China; 6 Department of Basic and Applied Neurobiology, Federal Medical Research Center for Psychiatry and Narcology, Moscow, Russian Federation; 7 Department of Psychology, University of the Free State, Turfloop, South Africa; 8 Department of Oncology, Radiology and Nuclear Medicine, Medical Institute of Peoples' Friendship University of Russia, Moscow, Russian Federation; 9 School of Management, Tianjin University of Traditional Chinese Medicine, Tianjin, China

**Keywords:** osteosarcoma, methotrexate, leukopenia, risk factor, nomogram

## Abstract

Purpose: To explore the trends of plasma drug concentration changes after high-dose methotrexate (MTX) treatment of osteosarcoma (OS), analyse the risk factors for leukopenia (LP) after MTX treatment, and establish a LP prediction nomogram.

Methods: A total of 35 OS patients at Tianjin Medical University Cancer Institute and Hospital between 2017 and 2021 were collected (the construction cohort). Another 12 OS patients between 2019 and 2021 in P.A. Hertsen Moscow Oncology Research Center were involved (the external validation cohort). Peripheral venous blood MTX concentration (C_MTX_) was monitored at 0h, 6h, 24h, 48h and 72h after MTX administration. The characteristics were collected: age, sex, body surface area, lesion site, pathological subtype, pathological fractures, American Joint Committee on Cancer (AJCC) clinical stage, MTX dose, tumour necrosis, Ki-67 index, erythrocyte count, haemoglobin count, white blood cell count, platelet count (PLT), alanine aminotransferase (ALT), aspartate aminotransferase (AST), bilirubin, albumin concentration, creatinine, alkaline phosphatase, and lactate dehydrogenase. Logistic regression analysis was used to determine the risk factors for LP occurrence. Significant factors were used to construct the prediction nomogram.

Results: A total of 128 MTX chemotherapy cycles from 35 OS patients were included. Female, Ki-67>20%, C_MTX_>112μmol/L at 6h, PLT, and AST were risk factors for post-chemotherapy LP occurrence. The LP prediction nomogram was created and validated.

Conclusions: Female, C_MTX_ at 6h, Ki-67 index, AST and PLT before MTX treatment were risk factors for LP in OS patients who received MTX treatment. The established nomogram can guide personalized LP prediction in OS patients receiving MTX chemotherapy.

## INTRODUCTION

Osteosarcoma (OS) is the most common primary malignant tumour of bone and has a bimodal age distribution; i.e., it often occurs in adolescents and elderly people. The global annual incidence rate is approximately 3 to 4 cases per million people [1]. Since the emergence of the neoadjuvant chemotherapy-surgery-adjuvant chemotherapy model, the 5-year event-free survival rate for OS patients without distant metastasis has reached 70% [2, 3]. Methotrexate (MTX) is a dihydrofolate reductase inhibitor. It can significantly reduce the synthesis of DNA, RNA and protein in tumour cells and inhibit tumour cell proliferation [4]. Currently, multidrug combination chemotherapy containing MTX has become the recommended chemotherapy regimen in international guidelines for the treatment of OS [5].

The recommended dose for the clinical MTX treatment of OS is 8-12 g/m^2^_._ Previous studies have shown that a plasma concentration of MTX (C_MTX_) ≥ 700 μmol/L for more than 6h can significantly increase nephrotoxicity, causing tubular degeneration and necrosis, delaying the excretion of MTX, which in turn can cause serious toxic reactions in multiple organs including the liver, bone marrow and gastrointestinal tract, and delaying the overall course of sequential chemotherapy. It can be fatal in severe cases [4, 6]. Previous studies have found that C_MTX_ at 48 h and 72 h after drug administration was higher than 1 μmol/L and 0.1 μmol/L, respectively, suggesting a delay in MTX metabolism and thus indicating an increased risk of adverse events and the need for targeted increases in detoxification doses and the frequency of alkalinization, hydration and leucovorin calcium [7].

Leukopenia (LP) is one of the most common complications of MTX treatment. A previous study reported that approximately 16.8% of patients have grade IV LP after MTX treatment [4]. Studies have shown that grade III-IV LP can significantly increase the risk of secondary infection in patients, causing pneumonia, endocarditis and urinary system infection and even death. Therefore, patients who have developed grade III and IV LP often need to be treated in isolation with close body temperature monitoring and supplemented with antibiotics and antifungal drugs to prevent the onset of infection, which increases medication risks and places a socio-economic burden on patients [8]. Previous studies that have analysed risk factors for the development of LP after MTX treatment are scarce, and there is a lack of credible predictive models, requiring urgent in-depth exploration.

In summary, the aim of this retrospective study was to establish C_MTX_ curves for 0 h, 6 h, 24 h, 48h and 72 h after MTX administration based on a single-center OS cohort, to analyse the trend of changes in C_MTX_, to explore the correlation between each C_MTX_ monitoring time point and the occurrence of LP, and to explore the best monitoring point for the early prediction of LP. Additionally, the clinical and pathological characteristics of patients were combined to analyse the risk factors for LP and establish a nomogram prediction model. This study will lay a foundation for the early prediction and prevention of LP after MTX treatment.

## RESULTS

### Clinical pathological characteristics of patients

**Figure 1:**
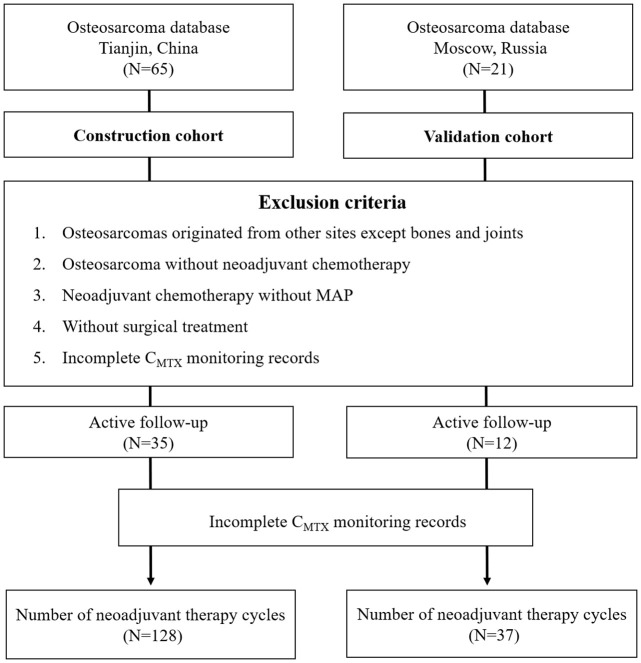
Flowchart for the selection of OS patients.

From January 2017 to December 2021, a total of 65 OS patients were recorded in Tianjin Medical University Cancer Institute and Hospital, Tianjin, China. Based on the inclusion and exclusion criteria, 35 OS patients were included in the construction cohort (19 males and 16 females with the median age of 16 years). A total of 128 cycles of MTX therapy were performed. Additionally, 21 OS patients from P.A. Hertsen Moscow Oncology Research Center between January 2019 to December 2021 were recorded, a total of 12 patients were included as the external validation cohort (7 males and 5 females with the median age of 16 years). A total of 37 cycles of MTX therapy were performed (Figure 1).

Among all OS lesions of the construction cohort, 19 were located in the distal femur, 12 were located in the proximal tibia, and a total of 4 were located in the proximal humerus, ilium, clavicle, and fibula. Regarding the pathological type of OS, 29 cases were osteogenic, 5 cases were chondrogenic, and 1 case was fibrogenic. Based on AJCC classification, 8 cases were grade IIA, 23 cases were grade IIB, and 4 cases were grade III. Three cases were accompanied by pathological fractures at the first diagnosis. The clinicopathological characteristics of the patients, who underwent a total of 128 chemotherapy cycles, are shown in Table 1.

**Table 1:** Baseline demographic and clinicopathological characteristics in the construction cohort and external validation cohort.

	**Construction cohort**				**Validation cohort**		
	**Non-LP**	**LP**	**p**		**Non-LP**	**LP**	**p**
N	60	68			11	26	
Age (years)	15 [14, 20]	16 [14, 19]	0.659		19.00 [12.00, 26.50]	16.00 [13.00, 26.50]	0.84
Sex			0.003				0.482
Male	43 (71.7)	30 (44.1)			8 (72.7)	14 (53.8)	
Female	17 (28.3)	38 (55.9)			3 (27.3)	12 (46.2)	
BSA (m^2^)*	1.85 [1.64, 1.98]	1.64 [1.50, 1.65]	<0.001		1.91 [1.80, 1.97]	1.66 [1.42, 1.97]	0.052
Location			0.956				0.51
Distal femur	35 (58.3)	41 (60.3)			8 (72.7)	14 (53.8)	
Proximal tibia	20 (33.3)	21 (30.9)			2 (18.2)	6 (23.1)	
Other	5 (8.3)	6 (8.8)			1 (9.1)	6 (23.1)	
Pathology			0.312				0.164
Osteogenic	49 (81.7)	53 (77.9)			10 (90.9)	16 (61.5)	
Chondrogenic	10 (16.7)	10 (14.7)			1 (9.1)	10 (38.5)	
Fibrogenic	1 (1.7)	5 (7.4)			-	-	
Fracture	11 (18.3)	7 (10.3)	0.293		11 (100.0)	26 (100.0)	NA
AJCC			0.579				0.04
IIA	12 (20.0)	15 (22.1)			0 (0.0)	4 (15.4)	
IIB	37 (61.7)	45 (66.2)			9 (81.8)	22 (84.6)	
III	11 (18.3)	8 (11.8)			2 (18.2)	0 (0.0)	
adMTX* (g)	14 [14, 16]	14.00 [12, 15]	0.004		15.00 [14.00, 16.00]	14.00 [12.00, 14.00]	0.052
Necrosis			0.664				0.333
< 90%	35 (58.3)	36 (52.9)			6 (54.5)	20 (76.9)	
> 90%	25 (41.7)	32 (47.1)			5 (45.5)	6 (23.1)	
Ki-67			0.001				0.333
< 20%	24 (40.0)	9 (13.2)			5 (45.5)	6 (23.1)	
> 20%	36 (60.0)	59 (86.8)			6 (54.5)	20 (76.9)	
Pre-chemotherapy RBC (×10^9^/L)	4.09 [3.75, 4.42]	3.84 [3.45, 4.23]	0.005		4.86 [4.62, 5.42]	3.20 [2.84, 3.56]	<0.001
HBG (g/L)	119 [108, 132]	112 [104, 123]	0.034		132.00 [120.00, 138.00]	112.00 [92.00, 125.75]	0.032
WBC (×10^9^/L)	5.60 [4.31, 7.21]	5.36 [4.25, 7.00]	0.793		6.24 [5.30, 7.24]	5.84 [4.33, 8.12]	0.778
PLT (×10^9^/L)	257 [216, 324]	231 [176, 284]	0.004		298.00 [254.00, 329.00]	156.50 [121.00, 205.00]	<0.001
ALT (U/L)	22 [11, 31]	18 [13, 27]	0.638		16.00 [9.50, 19.50]	21.50 [14.25, 39.00]	0.058
AST (U/L)	19.00 [16, 23]	20 [16, 28]	0.326		23.00 [16.00, 25.00]	42.00 [37.00, 56.75]	<0.001
TBL (μmol/L)	11.0 [6.9, 14.7]	9.3 [5.9, 12.5]	0.155		10.00 [8.60, 11.40]	7.05 [5.23, 13.78]	0.344
Protein (g/L)	41.7 [38.8, 44.0]	41.0 [38.4, 43.4]	0.562		42.40 [41.70, 46.45]	42.40 [38.73, 44.25]	0.213
sCr (μmol/L)	52 [44, 60]	54 [44, 57]	0.819		56.00 [46.50, 63.00]	51.50 [44.25, 60.75]	0.606
ALP (mmol/L)	163 [95, 233]	119 [85, 197]	0.326		203.00 [118.00, 296.50]	157.00 [108.00, 216.00]	0.207
LDH (IU/L)	165 [148, 196]	174 [132, 209]	0.994		260.00 [210.00, 320.50]	183.00 [112.00, 232.25]	0.056
0 h C_MTX_	945.33 [697.79, 1139.68]	952.63 [830.11, 1056.07]	0.652		812.30 [790.67, 926.10]	1308.91 [874.48, 1565.14]	0.026
6 h C_MTX_	88.66 [78.40, 111.52]	121.98 [111.90, 132.48]	<0.001		113.31 [89.84, 118.82]	86.28 [58.05, 107.73]	0.107
24 h C_MTX_	0.53 [0.34, 0.72]	1.25 [0.80, 1.73]	<0.001		0.78 [0.60, 0.99]	0.37 [0.29, 0.51]	0.001
48 h C_MTX_	0.07 [0.05, 0.11]	0.12 [0.06, 0.20]	0.001		0.08 [0.06, 0.20]	0.09 [0.05, 0.16]	0.618
72 h C_MTX_	0.02 [0.01, 0.03]	0.02 [0.01, 0.04]	0.705		0.03 [0.02, 0.04]	0.03 [0.02, 0.04]	0.666
Post-chemotherapy WBC (×10^9^/L)	3.67 [3.30, 4.23]	2.19 [1.96, 2.54]	<0.001		3.28 [2.80, 4.04]	1.99 [1.73, 2.36]	<0.001
adMTX* >14 g (%)	27 (45.0)	21 (30.9)	0.143		6 (54.5)	3 (11.5)	0.018
6h C_MTX_ >112 (%)	14 (23.3)	51 (75.0)	<0.001		6 (54.5)	6 (23.1)	0.138

*adMTX, average dose MTX; BSA, body surface area.

### Distribution of C_MTX_

**Figure 2:**
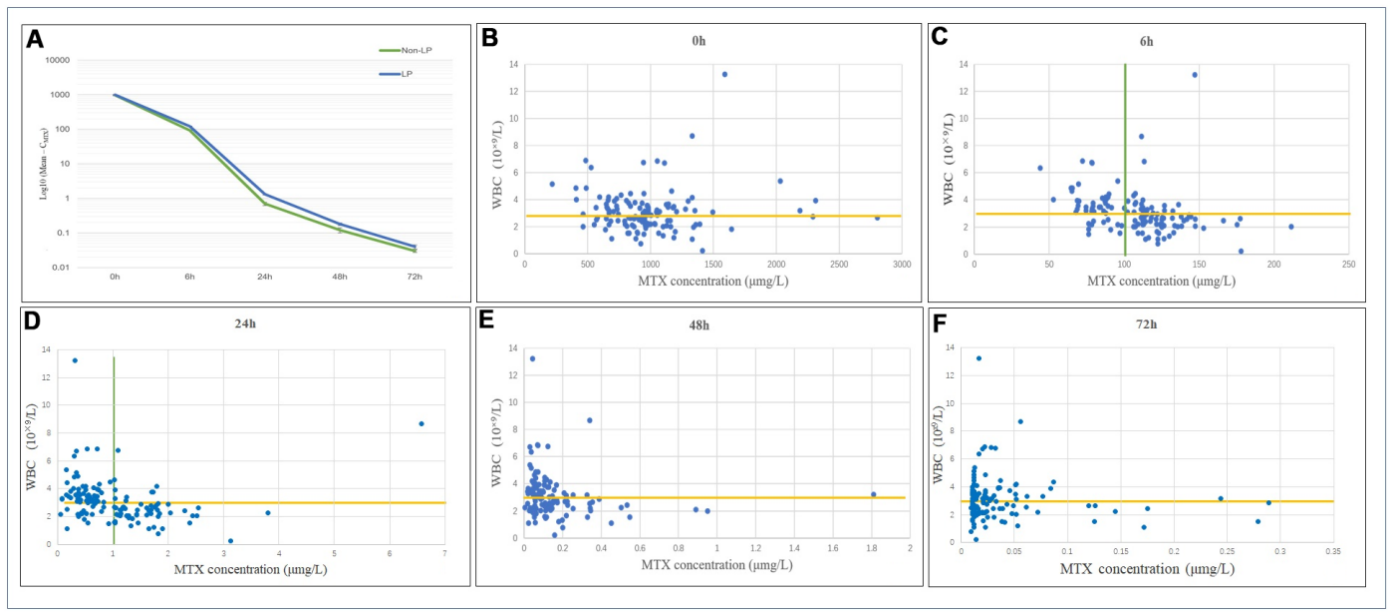
(**A**) Distribution and variation trends of C_MTX_. The overall change trend of C_MTX_ at different time points after MTX chemotherapy. LP: the leukopenia group; non-LP: the non-leukopenia group. (**B**–**F**) Relationship between C_MTX_ levels and LP at different time points. C_MTX_ concentration at 6h and 24h showed the highest sensitivity and specificity for LP prediction, which were 83.8% and 58.3%, 72.1% and 83.3%, respectively.

The total MTX doses in the LP and non-LP groups were 13.63±1.64 g and 14.48±1.44 g, respectively, with no significant difference between the 2 groups. Among the patients included in this study, the mean C_MTX_ levels immediately after 0 h and 6 h, 24 h, 48 h, and 72 h after MTX treatment were 980.05±373.53 μmol/L, 108.47±27.48 μmol/L, 1.02±0.84 μmol/L, 0.15±0.21 μmol/L, and 0.04±0.05 μmol/L, respectively. For patients in the post-chemotherapy LP group, the C_MTX_ levels at 0 h, 6 h, 24 h, 48 h and 72 h after MTX administration were 990.58±354.43 μmol/L, 121.99±24.86 μmol/L, 1.3±0.7 μmol/L, 0.18±0.18 μmol/L and 0.04±0.06 μmol/L, respectively. For patients in the post-chemotherapy non-LP group, the C_MTX_ levels at 0 h, 6 h, 24 h, 48 h and 72 h after MTX administration were 968.13±396.75 μmol/L, 93.15±21.76 μmol/L, 0.70±0.86 μmol/L, 0.12±0.23 μmol/L and 0.03±0.03 μmol/L, respectively. Six hours after MTX administration, the mean C_MTX_ value was 108.47±27.48 μmol/L. There was a significant difference in C_MTX_ at 6 h between the 2 groups (p<0.001). Twenty-four hours after MTX administration, the mean C_MTX_ was 1.02±0.84 μmol/L. There was a significant difference in C_MTX_ at 24 h between the 2 groups (p<0.001). The trend of the variation in C_MTX_ at different time points is shown in Figure 2A.

The relationship between C_MTX_ and postoperative WBC counts at different time points is shown in Figure 2B–2F. When the cut-off value for C_MTX_ was 100.0 μmol/L at 6 h, the sensitivity and specificity of predicting a WBC count below 3×10^9^/L after MTX administration were 83.8% and 58.3%, respectively. Among them, after 82 MTX treatments, C_MTX_ ranged from 100.26 to 211.49 μmol/L, and 57 cases of LP occurred; C_MTX_ was <100.0 μmol/L after the remaining 46 MTX treatments, and a total of 11 cases of LP occurred.

When the cut-off value for C_MTX_ was 1.0 μmol/L at 24 h, the sensitivity and specificity of predicting a WBC count below 3×10^9^/L were 72.1% and 83.3%, respectively. Among them, after 69 MTX treatments, C_MTX_ ranged from 0.05 to 0.94 μmol/L, and a total of 19 cases of LP occurred; C_MTX_ was >1.0 μmol/L after the remaining 59 MTX treatments, and a total of 49 cases of LP occurred. In this study, a total of 9 patients receiving MTX chemotherapy treatment had a C_MTX_ > 0.1 μmol/L after 72 h, suggesting delayed MTX excretion; among these patients, 8 cases of LP occurred.

**Figure 3:**
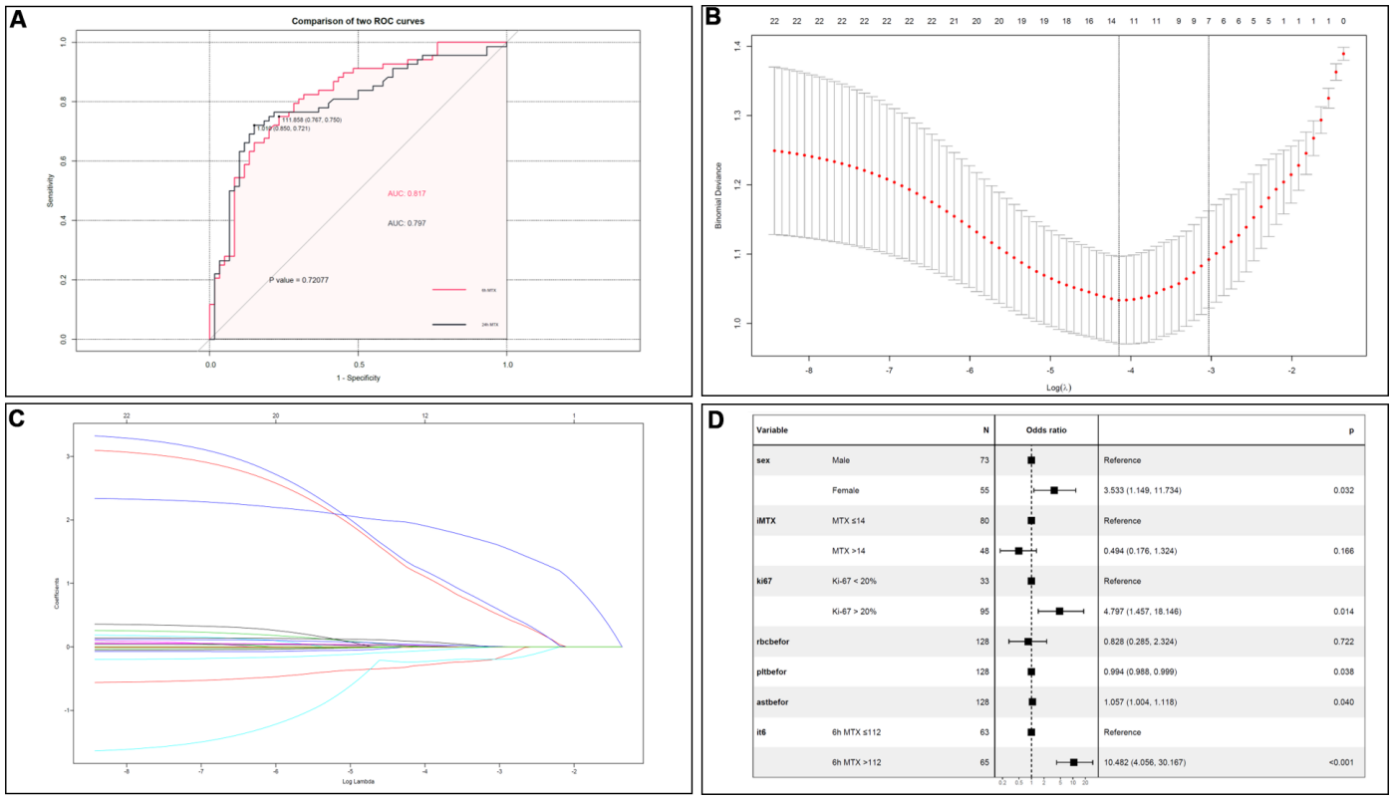
(**A**) Comparison of C_MTX_ between 6h and 24h. There was no significant difference on LP prediction between CMTX at 6h and 24h. (**B**) Cross validation plot for the penalty term. (**C**) LASSO coefficient profiles of LP-related factors. (**D**) Forest plots of the multivariate logistic regression analyses.

By calculating the area under the receiver operating characteristic (ROC) curve (AUC), it was found that when the C_MTX_ cut-off value was 112 μmol/L at 6 h, the sensitivity and specificity of predicting a WBC count below 3×10^9^/L after MTX administration were 75.0% and 76.7%, respectively, with an AUC of 0.817. When the C_MTX_ cut-off was 1.01 μmol/L at 24h, the sensitivity and specificity were 72.1% and 85.0%, respectively, and the AUC was 0.797. There was no significant difference between 6h and 24h (p=0.721). The ROC curves are shown in Figure 3A.

### Risk factors for LP after MTX administration

Based on the logistic multivariate analysis, females (OR=3.533, 95% CI 1.149–11.734, P=0.032), Ki-67 index > 20% (OR=4.797, 95% CI 1.457-18.146, P=0.014), average dose MTX >14 g (OR=0.494, 95% CI 0.176-1.324), C_MTX_ at 6h >112 μmol/L (OR=10.482, 95% CI 4.056–30.167, P<0.001), RBC (OR=0.828, 95% CI 0.285-2.324), PLT (OR=0.994, 95% CI 0.988–0.999, P=0.038), and AST concentration (OR=1.057, 95% CI 1.004-1.118, P=0.040) were risk factors for LP after chemotherapy. All the indicators were shown in Figure 3B–3D.

### Establishing a nomogram to predict the risk of LP

**Figure 4:**
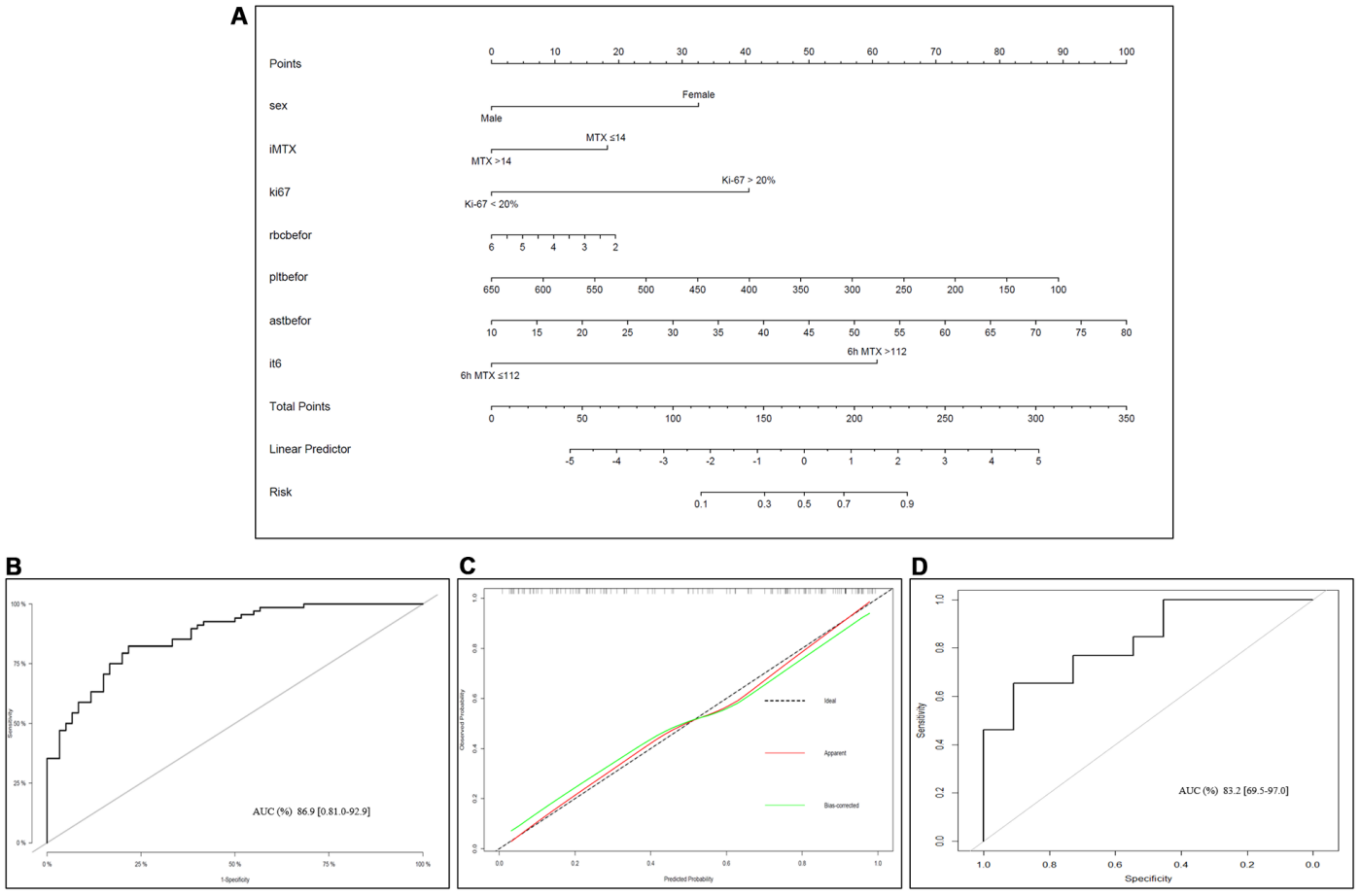
**Nomogram of LP prediction after MTX chemotherapy in OS.** (**A**) Nomogram model for predicting the occurrence of LP. The clinical and pathological characteristics of the patients were placed on the axis with the variables in the nomogram. The score for each variable was marked. By calculating the sum of these values and marking them on the total score line, the probability of LP in OS patients after receiving MTX treatment was obtained. (**B**) An ROC curve was used to evaluate the sensitivity and specificity of the nomogram in predicting the occurrence of LP after MTX treatment (the AUC was 86.9%, the specificity was 81.0%, and the sensitivity was 92.9%). (**C**) Evaluation of the calibration curves for the nomogram for predicting the occurrence of LP after MTX treatment. The calibration curve indicated that the predicted LP values of the nomogram were in good agreement with the observed results; the x-axis represents the predicted probability of LP, and the y-axis represents the actual probability of LP. The black dashed line represents the perfect prediction probability of the ideal model, the red solid line represents the nomogram prediction plot, and the green solid line represents the corrected bootstrap. An accurately calibrated nomogram curve is close to an ideal 45° straight line. (**D**) ROC curve in the external validation cohort.

Based on multivariate logistic regression model results, we selected the following variables to construct prediction spectra of the occurrence of LP in OS patients after MTX treatment: sex, adMTX, Ki-67, C_MTX_ at 6h, RBC, PLT and AST before neoadjuvant chemotherapy (Figure 4A). The nomogram showed good accuracy in predicting the risk of LP, with a C-index of 0.869 (95% CI: 0.810-0.929). The AUC of this prediction model was 86.9%, suggesting that the model has a relatively satisfactory prediction ability (Figure 4B). In addition, the calibration curves for the nomogram prediction model were all close to the 45-degree line, and the predicted values were in good agreement with the observed values (Figure 4C).

### External validation of the prediction model

Detailed information on the clinicopathological characteristics of the external validation cohort was shown in Table 1. As shown in Figure 4D, the AUC of the predictive nomogram based on the external validation cohort were 0.8322 (95% CI: 0.695-0.970).

## DISCUSSION

OS is a highly malignant tumour of mesenchymal origin originating from the mesoderm. Rosen et al. stated that after the emergence of the MTX-based chemotherapy model, treatment effects have progressed substantially [9]. However, the severe toxicity of MTX limits its wide application, and its metabolism can vary more than 10-fold among individuals. Even under the standard adjuvant treatment model recommended by the National Comprehensive Cancer Network (NCCN), 24% of patients still develop LP. The toxicity of MTX has become one of the major bottlenecks of sequential chemotherapy regimens, restricting OS treatment effects [6].

There were large individual variations in C_MTX_ at various time points [10]. To prevent the occurrence of peri-treatment adverse reactions to and complications of MTX, the C_MTX_ at 0 h after the treatment of OS with high-dose MTX should be controlled between 700 and 1000 μmol/L, and the average C_MTX_ levels at 24 h, 48 h, and 72 h should be lower than 10 μmol/L, 1 μmol/L, and 0.1 μmol/L, respectively. C_MTX_ levels exceeding the above ranges indicate delayed MTX excretion, suggesting the occurrence of toxic reactions in the liver, kidney, and heart [11]. The average C_MTX_ observed at 0 h in this study was 980.05±373.53 μmol/L. It was 108.47±27.48 μmol/L at 6 h and 1.02±0.84 μmol/L at 24 h. At 48 h and 72 h, the C_MTX_ levels were significantly lower than those reported in other studies [7]. This finding may be related to detoxification with adequate alkalization, hydration and standard leucovorin calcium.

LP is one of the most common complications after MTX treatment. Early detection and timely treatment of LP will significantly reduce the incidence of secondary infections. C_MTX_ at 48 h is often used clinically as a predictive time point for the development of hepatotoxicity and nephrotoxicity. However, no in-depth research has been done on whether LP occurs [11]. This study found that the when the C_MTX_ levels at 6 h and 24 h were greater than 112 μmol/L and 1 μmol/L, respectively, the incidence of LP increased significantly. By calculating the AUC, it was found that there was no significant difference in the predictive performance between these 2 time points (P>0.05). Compared with that at 24 h, C_MTX_ at 6 h provided earlier warning of LP and guided the necessary prophylactic treatment (granulocyte colony-stimulating factor or mecapegfilgrastim), thus effectively preventing potentially life-threatening secondary infections. Mecapegfilgrastim is a new agent in the family of long-acting granulocyte colony-stimulating factors (G-CSF), and is intended for use in patients with non-myeloid malignancies receiving myelosuppressive anti-cancer therapy associated with a clinically significant incidence of leucopenia and febrile neutropenia. The efficacy and safety of mecapegfilgrastim, using a regimen of once-per-cycle injection of 100-μg/kg or a fixed 6-mg dose [12, 13].

Previous studies found that being female was an independent risk factor for the development of LP after MTX treatment for OS [14]. Currently, there are few studies on the effect of sex on MTX metabolism, and the results are controversial. However, the analysis in this study showed that the incidence of severe delayed MTX excretion was significantly higher in female OS patients than in male OS patients [12]. The poor metabolic function of the liver in female patients may be an important factor causing the reduction in secondary LP [14]. Therefore, it is necessary to increase the degree of hydration and alkalization and establish a more stringent monitoring system for MTX treatment in female OS patients who have poor liver metabolic function. This study also found that being female was a risk factor for LP, a finding that was consistent with those of previous reports.

This study, for the first time, revealed that the occurrence of LP was closely related to the Ki-67 index and that patients with Ki-67>20% were more prone to LP. The reasons may be that cells in the G1 phase mainly express Ki-67 [15], while MTX acts mostly on cells in the S phase, with a weak ability to kill cells in the G1 phase [16]. Therefore, the higher is the Ki-67 index, the poorer is the ability of MTX to kill tumour cells, leading to an increase in MTX content in serum and the development of LP. Thus, an increased Ki-67 index is an independent risk factor for the development of LP and can be used for the stratified management of patients and screening patients prone to LP.

In addition, our team found that the occurrence of LP in OS patients after MTX treatment was closely related to AST concentration. Previous studies have noted that when MTX is metabolized in the liver, it can not only cause damage to liver cells but can also cause reversible chemical hepatitis in 60% of patients and hyperbilirubinemia in 25% of patients [4, 17]. According to statistics, 15-50% of patients have different degrees of increase in serum AST and/or ALT concentrations after receiving MTX treatment [16]. Therefore, OS patients with poor liver function may develop liver toxicity after receiving MTX treatment, resulting in hepatocyte damage and the release of AST, and MTX that has not been completely metabolized by the liver will cause bone marrow suppression, thereby causing LP. Therefore, liver function should be improved as much as possible during the MTX peri-treatment period to avoid secondary LP. Additionally, the results of this study suggested that patients with thrombocytopenia before MTX treatment were more prone to develop LP. Patients with thrombocytopenia should be closely observed, and necessary intervention and correction should be performed to prevent the occurrence of LP after MTX treatment.

This study investigated the risk of LP in OS patients during the MTX peri-treatment period from the perspective of retrospective observation and analysis. Logistic regression analysis was used to determine the risk factors for the development of LP. The developed nomogram model can be a potentially effective tool for early screening for the occurrence of LP. This study has certain limitations. First, this was a single-center retrospective study with a small sample size. The overfitting of the factors affecting LP occurrence might be inevitable. Meanwhile, we didn’t make a distinction between adolescent and adults. Future studies will be needed. Second, this study did not explore in depth the correlation between C_MTX_ at each monitoring point and the LP grades after chemotherapy. This correlation can provide an early warning of the degree of bone marrow suppression after MTX treatment, and leukocyte-promoting drugs can be used to prevent the occurrence of LP before MTX treatment. In addition, cancer rehabilitation treatment before chemotherapy can reduce appetite loss, nausea, vomiting, and bone marrow suppression-related complications caused by chemotherapy drugs to a certain extent. Therefore, in future studies, the positive impact of tumour rehabilitation on OS chemotherapy should be thoroughly explored to reduce the possibility of adverse events in OS patients during chemotherapy. This study revealed the trend of changes in the C_MTX_ of OS patients after receiving MTX chemotherapy, confirmed the feasibility of predicting the occurrence of LP based on the C_MTX_ levels at 6 h and 24 h after the administration of MTX, and explored a series of risk factors for LP (female, C_MTX_ at 6 h, Ki-67 index, AST concentration and PLT before MTX treatment). Based on the above clinicopathological characteristics, this study established a nomogram to predict the risk of LP in OS after receiving high-dose MTX and to ensure the safety of high-dose MTX to improve the long-term survival and quality of life of OS patients.

## MATERIALS AND METHODS

### Inclusion and exclusion criteria

We retrospectively analysed 65 patients with OS who received comprehensive treatment between January 2017 and December 2020 at Tianjin Medical University Cancer Institute and Hospital, Tianjin, China. Among these patients, 35 patients were assigned to the construction cohort. Additionally, 21 patients with OS diagnosed between January 2019 to December 2021 in P.A. Hertsen Moscow Oncology Research Center, Moscow, Russia were recorded, and in total of 12 patients were included as the external validation cohort to validate the constructed model.

The inclusion criteria were as follows: patients with primary OS confirmed by pathological diagnosis and who received 2 complete preoperative rounds of MAP (MTX, cisplatin and doxorubicin) sequential neoadjuvant chemotherapy; routine blood test results before each round of chemotherapy suggesting no significant bone marrow function; and patients with detailed data.

The exclusion criteria were as follows: patients with no clear pathological diagnosis and/or multiple malignancies; OS patients who did not receive neoadjuvant chemotherapy; patients with parosteal OS or secondary OS; patients with bone marrow dysfunction suggested by routine blood results before chemotherapy; and patients with missing data.

Based on the clinical and pathological characteristics of the patients, the following characteristics were included in this study: age, sex, body surface area, lesion site, pathological subtype, presence of pathological fractures, American Joint Committee on Cancer (AJCC) clinical stage, average dose MTX, tumour necrosis rate, Ki-67 index, pre-chemotherapy erythrocyte count, haemoglobin count, white blood cell (WBC) count, platelet count (PLT), alanine aminotransferase (ALT) concentration, aspartate aminotransferase (AST) concentration, total bilirubin concentration, albumin concentration, serum creatinine concentration, alkaline phosphatase concentration, lactate dehydrogenase (LDH) concentration, C_MTX_ at various monitoring time points during chemotherapy, and WBC count within 1 week after chemotherapy.

All patients’ guardians in this study were informed, and a detailed data collection sheet was filled. All the information gathered from the patients or their caregivers.

### High-dose MTX treatment regimen and C_MTX_ monitoring

Patients were hydrated and alkalized 12 h before high-dose MTX treatment: 500 mL of 0.9% NaCl + 500 mL of 0.9% NaCl + 250 mL of 5% NaHCO_3_. Patients received continuous administration of the following during MTX administration: (1) 500 mL of 5% NaCl + 500 mL of 0.9% NaCl + 10 mL of 15% KCl; (2) 500 mL of 5% NaHCO_3_; (3) 2 mg of vincristine + 50 mL of 0.9% NaCl; (4) continuous administration of 1000 mL of 0.9% NaCl + 8~12 g/m^2^ MTX for 5 h; and (5) 100 mL of 0.9% NaCl + 5 mg of tropisetron, 250 mL of 0.9% NaCl + 200 mg of vitamin B6, 100 mL of 0.9% NaCl + 1 mg of dexamethasone, and 100 mL of 0.9% NaCl + furosemide; the total fluid volume ranged from 3200 to 4000 mL, depending on each patient’s body mass index. The urine volume within 24 h was recorded, and the urine pH was measured to maintain the urine pH at 7-9. After the completion of MTX administration, hydration and alkalization treatment was continued for 2 days. During the entire MTX chemotherapy period, the patients received oral administration of NaHCO_3_ (1.0 g, tid) and allopurinol (200 mg, tid).

Two millilitres of blood were drawn from the peripheral venous at 0, 6, 12, 24, 48 and 72 h after MTX infusion. Blood samples were centrifuged at 3000g for 5 min at room temperature in tubes without anticoagulant. MTX concentration was measured using fluorescence polarization immunoassay (TDX, ABBOTT, USA) with a quantification limit of 0.01 μmol/L. Leucovorin calcium and diuretic detoxification therapy was started at 6 h. The leucovorin calcium detoxification regimen was as follows: intramuscular injection of 12-15 mg/m^2^ (q6h, d1 to d3), and then, individualized leucovorin calcium detoxification therapy was performed based on C_MTX_ until peripheral blood concentration of MTX was below 0.05 μmol/L, after which detoxification treatment was stopped [18].

### Toxicity analysis and statistical analysis

In this study, routine blood tests, liver and kidney function tests, and electrocardiograms were performed before MTX treatment, and routine blood tests and liver and kidney function were monitored within 1 week after sequential chemotherapy. Post-chemotherapy LP was defined as a WBC count<3.0×10^9^/L from day 1 to week 1 after chemotherapy [19–21].

Data are presented as frequencies (percentages) for categorical variables and medians (interquartile ranges [IQRs]) for continuous variables. Differences between groups were assessed by univariate analyses, specifically the chi-squared test for categorical variables and the Kruskal-Wallis test for continuous variables, respectively. The patients were divided into a normal group and an LP group. Cut-off values for C_MTX_ were determined by receiver operating characteristic (ROC) curve analysis. To further narrow the scope of the candidate explanatory variables, we adopted the least absolute shrinkage and selection operator (LASSO) logistic regression to select variables. We utilized ten-fold cross-validation and select the optimal penalty term, lambda.1se, which represents largest lambda that is still within one standard error of the minimum binomial deviance. Then, the multivariate logistic regression analysis was used to assess the selected covariates for the development of LP, and the odds ratio (OR) and 95% confidence interval (CI) of relevant indicators were calculated. A clinical prediction nomogram to assess the prognostic model of LP was constructed based on the results from the final multivariable logistic regression. In addition, the nomogram was subjected to bootstrapping validation (1000 bootstrap resamples) for internal validation to assess predictive accuracy. R 3.6.3 software was used for data processing and analysis. The validity and accuracy of the proposed models were further tested by the external validation cohort. P<0.05 was considered statistically significant.

### Availability of data and material

The datasets used and/or analysed during the current study are available from the corresponding author on reasonable request.

### Ethics approval

The present study complied with the 1964 Helsinki Declaration and its later amendments or comparable ethical standards, and the Research Ethics Board of the Tianjin Medical University Cancer Institute and Hospital approved the study.
